# Determinants of Delivery Mode Preferences and Decision‐Making Among Jordanian Women: A Cross‐Sectional Study

**DOI:** 10.1155/jp/8395387

**Published:** 2025-12-12

**Authors:** Hala Bawadi, Zaid Hamdan, Nagham Abu Shaqra, Maher Maaitah, Abdelmanie Suleimat, Asma Basha, Shawqi Saleh, Mazen El -Zibdah, Raja Khater, Ahmad Abdulla

**Affiliations:** ^1^ Department of Maternal and Child Health, School of Nursing, The University of Jordan, Amman, Jordan, ju.edu.jo; ^2^ Community and Mental Health Department, Faculty of Nursing, Jordan University of Science and Technology, Irbid, Jordan, just.edu.jo; ^3^ Office of Population and Family Health, USAID, Amman, Jordan; ^4^ Obstetrics and Gynecology Department, Jordanian Royal Medical Services, Amman, Jordan, jrms.mil.jo; ^5^ Ministry of Health, Amman, Jordan, behdasht.gov.ir; ^6^ The University of Jordan and Jordan University Hospital, Amman, Jordan; ^7^ The University of Jordan, Amman, Jordan, ju.edu.jo; ^8^ Obstetrics and Gynecology Consultant at Private Sector, Amman, Jordan; ^9^ USAID Health Services Quality Accelerator Activity, University Research Company, Amman, Jordan; ^10^ USAID Health Services Quality Accelerator Activity, Amman, Jordan

**Keywords:** cesarean section, Jordan, knowledge and belief, maternal health, mode of delivery decisions, vaginal birth

## Abstract

**Background:**

The birthing process presents women with both physical and emotional challenges. In recent years, there has been a notable global rise in cesarean section (CS) rates—particularly elective CS—including in Jordan. Numerous personal, cultural, and healthcare system–related factors contribute to women′s increasing preference for CS over vaginal delivery. This study explores the factors influencing Jordanian women′s knowledge, beliefs, and preferences regarding mode of delivery and their involvement in related decision‐making.

**Methods:**

A cross‐sectional study was conducted among Jordanian women in their second or third trimester of pregnancy, who were either primiparous or para‐one. A structured self‐administered questionnaire was used to collect data from a sample of 378 participants, encompassing demographic details, knowledge, preferences, beliefs, and decision‐making related to delivery mode.

**Results:**

Most participants (57.2%) were between 25 and 34 years of age, and 63.0% were in their third trimester. Doctors (81.5%) and nurses (39.6%) were the most frequently cited sources of information about maternal health. The average knowledge score was 71.4%, with higher knowledge levels observed among women receiving prenatal care at university‐affiliated or private facilities. Preference leaned more strongly toward vaginal delivery over CS. Belief scores averaged 73.3%, though several misconceptions persisted. Decision‐making scores were moderate, with higher involvement observed among women with better knowledge and more positive preferences toward vaginal delivery. Regional disparities were evident, with women in the southern region demonstrating greater decision‐making participation than those in central areas.

**Implications:**

The findings underscore the importance of enhancing prenatal education and healthcare counseling tailored to women′s regional and educational contexts. Increasing awareness of the benefits and risks associated with both CS and vaginal birth can support informed, autonomous decisions and improve maternal care outcomes across Jordan.

## 1. Introduction

Childbirth remains a profound physical and psychological experience, deeply shaped by cultural, medical, and personal factors. In recent decades, a growing trend has emerged globally: More women are exercising autonomy over their birth plans and opting for cesarean section (CS) deliveries, even in the absence of clinical indications. This shift toward elective cesarean delivery is evident across countries of varying economic status, suggesting that the phenomenon is influenced by more than just healthcare infrastructure [1, 2].

Although cesarean delivery is a vital intervention in situations where vaginal birth poses significant risks, the increasing tendency to perform CS without clear medical need has raised concerns. This issue is especially salient in low‐ and middle‐income countries (LMICs), where cesarean decisions may reflect personal or provider preferences, institutional pressures, or sociocultural beliefs rather than evidence‐based clinical criteria [3, 4]. Such practices may expose women to unnecessary surgical risks, increased healthcare costs, and complications in future pregnancies [5].

According to the World Health Organization (WHO) [6], the global average rate of cesarean delivery has reached approximately 21%, far exceeding the recommended threshold of 10%–15% necessary to address medical needs. In Jordan, this trend mirrors international patterns. The Jordan Population and Family Health Survey (2017–2018) reported a national CS rate of 26%, reflecting a significant increase over previous years and raising policy concerns regarding maternal health practices [7].

Several interconnected factors have contributed to this rise, including variability in provider practice patterns, fear of malpractice litigation, and systemic organizational dynamics within health systems [8, 9]. Additionally, women may perceive cesarean birth as more convenient, less painful, or safer, often influenced by anecdotal narratives, social media, or prior negative childbirth experiences [10, 11]. In Jordan, physicians frequently attribute the rise in elective CS to increased maternal requests, reflecting shifting societal attitudes toward childbirth [2].

These developments underscore the urgent need to examine the underlying drivers of delivery mode preferences among women, especially in contexts where rising CS rates may not align with improved maternal or neonatal outcomes. This study is aimed at exploring women′s knowledge, preferences, and autonomy in deciding their mode of delivery. Specifically, it investigates the sociocultural and healthcare‐related factors that influence their choices and assesses how informed and empowered women feel in navigating childbirth decisions.

## 2. Methods

### 2.1. Study Design

A descriptive cross‐sectional design was employed to assess women′s knowledge, preferences, beliefs, and decision‐making processes regarding modes of childbirth. Data were collected using a structured questionnaire developed by the research team in collaboration with experts in maternal health. The instrument was informed by a comprehensive review of current literature and adapted to suit the sociocultural context of Jordan.

### 2.2. Instrumentation

The questionnaire consisted of five sections: demographics and obstetric history (21 items), knowledge about modes of delivery (11 items), preferences for vaginal versus cesarean birth (nine items), beliefs regarding childbirth methods (12 items), and decision‐making dynamics—tailored separately for primiparous women and para‐one women (10 items each).

Face and content validity were established through an expert evaluation by three maternal care specialists, ensuring cultural relevance and conceptual alignment with existing research. Internal consistency reliability was assessed for each subscale using Cronbach′s alpha, with all values exceeding 0.81, indicating good to excellent reliability.

### 2.3. Study Setting and Sample

Participants were recruited from a diverse mix of healthcare institutions across Jordan, including five maternity wards in tertiary hospitals and five public and private maternal health centers. Recruitment occurred between October 2024 and February 2025. Eligible participants were Jordanian women attending antenatal care during their second or third trimester, classified as either primiparous or para‐one. Women with high‐risk pregnancies or scheduled CSs for medical reasons were excluded from the study.

A sample size calculation was performed based on Cochran′s formula for cross‐sectional designs, targeting a 95% confidence level and a 5% margin of error. The required minimum sample was estimated at 350 participants. However, to enhance representativeness and account for possible nonresponse or incomplete data, a total of 378 participants completed the questionnaire and were included in this analysis. Recruitment used a convenience sampling approach, and data collection was supported by trained research assistants who provided clarification when needed.

### 2.4. Ethical Considerations

The study protocol received ethical approval from the Institutional Review Boards (IRBs) of all participating facilities. Additional permissions were obtained from the relevant health directorates and administrative bodies overseeing the selected institutions. Informed consent was obtained from all participants, and confidentiality and voluntary participation were assured throughout the research process.

## 3. Data Analysis

All data analyses were conducted using SPSS Version 27. Descriptive statistics were employed to summarize participant characteristics and study variables. Continuous variables were presented as means and standard deviations, whereas categorical variables were summarized using frequencies and percentages. To identify factors associated with women′s knowledge about vaginal delivery, preferences regarding CS, and underlying beliefs toward childbirth, stepwise multiple regression with backward elimination was applied. This approach allowed the retention of only statistically significant predictors within each model. In examining predictors of decision‐making processes among both primiparous and para‐one women, one‐way analysis of variance (ANOVA) was initially used to assess differences across participant subgroups based on sociodemographic characteristics. Variables found to be significant in the ANOVA were then incorporated into regression models alongside theoretically derived predictors, including knowledge levels, delivery preferences, and belief scores. Prior to running the regression analyses, all relevant assumptions were tested to ensure model validity. These included evaluating the linearity of relationships, the normal distribution and homoscedasticity of residuals, and checking for multicollinearity among independent variables. Statistical significance was determined using a two‐tailed approach, with a *p* value of less than 0.05 considered indicative of a significant association.

## 4. Results

### 4.1. Sample Characteristics

The final analytical sample included 378 Jordanian women in their second or third trimester of pregnancy. Table 1 provides an overview of participant demographics and obstetric history. The majority (56.6%) were between the ages of 25 and 34 years, with a mean age at marriage of 24.1 years (SD = 4.2). Approximately 45% of participants had been married for 1–3 years, whereas nearly two‐thirds (62.2%) held a bachelor′s degree. Regarding their husbands′ education levels, just over half (51.4%) had completed undergraduate studies. Most participants resided in urban areas (65.6%), with regional representation distributed across the northern (43.3%), central (41.4%), and southern (15.3%) parts of the country.

**Table 1:** Characteristics for the total sample (*n* = 378).

**Variable**	**Overall**
Age (%)	
< 18	5 (1.4)
18–24	93 (24.5)
25–34	216 (57.2)
35–44	55 (14.6)
45–49	9 (2.3)
Age at marriage (mean (SD))	24.1 (4.2)
Years married (%)	
< 1	38 (10.2)
1–3	174 (46.1)
4–6	83 (22.0)
7–9	25 (6.7)
> 9	56 (15.0)
Wife education level (%)	
Elementary school or less	22 (5.8)
High school	91 (24.1)
Undergraduate	242 (63.9)
Postgraduate	24 (6.2)
Husband education level (%)	
Elementary school or less	21 (5.6)
High school	146 (38.4)
Undergraduate	192 (50.7)
Postgraduate	21 (5.3)
Residence region (%)	
Middle	154 (40.7)
North	167 (44.2)
South	57 (15.0)
Residence place = urban (*%*)	248 (65.5)
Wife employment status (%)	
Unemployed	201 (53.5)
Nonprofit private	4 (0.9)
Public	120 (31.7)
Private	53 (13.9)
Husband employment status (%)	
Unemployed	28 (7.4)
Nonprofit private	6 (1.4)
Public	246 (65.5)
Private	98 (25.7)
Pregnancy age in weeks = third trimester (*%*)	238 (63.0)
Number of pregnancies (%)	
One	180 (47.7)
Two	122 (32.2)
> 2	76 (20.1)
Number of births = 1 (*%*)	202 (53.5)
Number of miscarriages before 24 weeks (%)	
0	275 (73.1)
1	70 (18.3)
> 2	33 (8.6)
Stillbirths after 24 weeks = yes (*%*)	25 (6.5)
Infertility issues = yes (*%*)	48 (12.5)
High − risk pregnancy = yes (*%*)	79 (20.6)
Previous birth = yes (*%*)	200 (52.8)
Insurance coverage type (%)	
Public	172 (45.4)
Military	96 (25.7)
University	28 (7.4)
Private	49 (13.0)
No insurance	33 (8.6)
Prenatal care location (%)	
Public	112 (30.1)
Military	39 (10.2)
University	34 (8.8)
Private	67 (17.6)
Multiple	126 (33.3)
Planned hospital for delivery (%)	
Public	152 (40.3)
Military	70 (18.3)
University	67 (17.6)
Private	78 (21.1)
Nonprofit	11 (2.8)

A substantial proportion of the women (53.5%) were unemployed, whereas their spouses were predominantly employed in the public (65.6%) or private (22.5%) sectors. Family income levels varied, with roughly one‐third (33.2%) reporting monthly household income in the 401–620 Jordanian Dinar range, followed by those earning less than 400 JD (25.1%), and 15.1% reporting income between 621 and 832 JD.

Nearly half of the participants (47.8%) were pregnant for the first time, and 62.9% were in their third trimester. The majority (52.4%) had previously given birth once. The rate of reported miscarriage before 24 weeks was relatively low, with 73.0% reporting no previous losses. A history of stillbirth after 24 weeks was noted in 6.5% of cases, and 12.4% of women indicated a history of infertility, whereas 20.8% identified themselves as having a high‐risk pregnancy.

Regarding infertility treatments, 87.0% had not undergone any form of intervention. Among those who did, in vitro fertilization was the most reported method (6.7%), followed by the use of fertility medications (5.5%) and intrauterine insemination (3.5%). Surgical intervention was the least common (1.2%).

**Figure 1:**
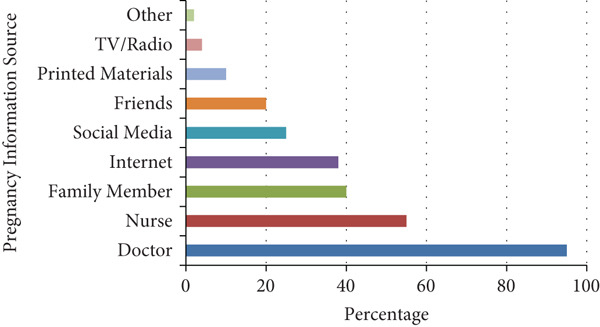
Source of information about motherhood and pregnancy (*n* = 432).

Figure 1 illustrates the most trusted sources of information on maternal health. Physicians were reported as the leading source (81.5%), followed by nurses (39.6%), family members (31.0%), internet‐based platforms (30.6%), and social media (21.5%). Other sources included friends, printed educational materials, television, and radio.

### 4.2. Knowledge, Preferences, Beliefs, and Decision‐Making Regarding Childbirth

The participants demonstrated a relatively high level of knowledge about delivery methods, with a mean knowledge score of 71.4%. The average preference score for normal vaginal delivery was 81.0% (24.3 out of 30), whereas preference for CS delivery was lower, averaging 59.3%. Belief scores regarding delivery methods showed a moderate to high alignment with evidence‐based perspectives, with an overall average of 73.3%.

Regarding decision‐making autonomy, primiparous women had a mean score of 70.0%, whereas para‐one women averaged 70.7%, indicating a moderate level of involvement in the decision‐making process. Across all domains, standard deviations were relatively low, suggesting consistent patterns in knowledge, preferences, and beliefs among the participants.

### 4.3. Prediction of Knowledge Regarding Preferred Mode of Delivery

To explore factors associated with women′s knowledge about childbirth methods, a multiple regression analysis was conducted. As shown in Table 2, regional differences emerged as significant predictors. Compared with women residing in the central region (the reference group, which includes the capital and is assumed to have the most comprehensive healthcare infrastructure), women in the northern region exhibited higher knowledge scores, with an average increase of 2.33 points (95% CI: −3.72 to −0.93; *p* = 0.001). Conversely, those in the southern region demonstrated significantly lower knowledge levels, with a mean difference of −7.64 points (95% CI: −9.62 to −5.65; *p* < 0.001).

**Table 2:** Prediction of knowledge regarding preferred mode of delivery among Jordanian women (*n* = 378).

**Variable**	**Estimate (95% CI)**	**p** **value**
Residence region		
Middle	Reference	Reference
North	2.33 (−3.72, −0.93)	0.001
South	−7.64 (−9.62, −5.65)	< 0.001
High‐risk pregnancy, yes	2.4 (0.89, 3.92)	0.002
Prenatal care location		
Public	Reference	Reference
Military	0.09 (−2.2, 2.37)	0.94
University	5.32 (2.79, 7.84)	< 0.001
Private	2.33 (0.33, 4.32)	0.02
Multiple	2.93 (1.24, 4.62)	< 0.001

Women identified as having high‐risk pregnancies were significantly more knowledgeable, with scores exceeding their counterparts by 2.4 points (95% CI: 0.89–3.92; *p* = 0.002). Access to university‐affiliated prenatal care was also a strong predictor of enhanced knowledge, contributing to a 5.32‐point increase (95% CI: 2.79–7.84; *p* < 0.001) when compared with those attending public health clinics. Those receiving care in private facilities reported significantly higher knowledge levels as well, with a difference of 2.33 points (95% CI: 0.33–4.32; *p* = 0.02). Similarly, seeking care from multiple locations was associated with better knowledge, increasing scores by 2.93 points (95% CI: 1.24–4.62; *p* < 0.001). In contrast, military health facilities did not significantly contribute to knowledge prediction (estimate = 0.09; 95% CI: −2.20 to 2.37; *p* = 0.94).

### 4.4. Prediction of Preference Toward Normal Vaginal Delivery

Regression analysis was performed to assess predictors of women′s preference toward normal vaginal delivery. Women residing in the southern region reported significantly lower preference scores compared with those in the central region (reference group), with an estimated difference of −1.35 points (95% CI: −2.29 to −0.41; *p* = 0.005). No significant difference in preference scores was found between women living in the northern region and those in the middle (*p* = 0.78) Table 3.

**Table 3:** Prediction of preference toward normal delivery among Jordanian women (*n* = 378).

**Variable**	**Estimate (95% CI)**	**p** **value**
Residence region		
Middle	Reference	Reference
North	0.1 (−0.58, 0.78)	0.78
South	−1.35 (−2.29, −0.41)	0.005
Insurance coverage		
Public	Reference	Reference
Military	−0.82 (−1.6, −0.05)	0.04
University	−1.74 (−2.98, −0.5)	0.006
Private	−0.26 (−1.23, 0.72)	0.61
No insurance	−1.41 (−2.57, −0.26)	0.02

Insurance coverage was also a significant factor influencing delivery preference. Women covered by military insurance had significantly lower preference scores toward vaginal delivery by 0.82 points (95% CI: −1.6 to −0.05; *p* = 0.04), and those with university insurance by 1.74 points (95% CI: −2.98 to −0.5; *p* = 0.006), compared with those with public insurance. Similarly, women with no insurance coverage exhibited lower preference scores by 1.41 points (95% CI: −2.57 to −0.26; *p* = 0.02). However, private insurance did not significantly differ from public coverage in its effect on preference scores (*p* = 0.61).

### 4.5. Prediction of Preference Toward CS

The analysis also examined factors influencing women′s preference for CS. Compared with the reference group of women aged < 18 years, those aged 18–24 years and 35–44 years were significantly more likely to prefer CS, with increases of 5.47 (95% CI: 0.91–10.03; *p* = 0.02) and 5.16 points (95% CI: 0.43–9.9; *p* = 0.03), respectively Table 4.

**Table 4:** Prediction of preference toward CS among Jordanian women (*n* = 378).

**Variable**	**Estimate (95% CI)**	**p** **value**
Age		
< 18	Reference	Reference
18–24	5.47 (0.91, 10.03)	0.02
25–34	3.35 (−1.21, 7.9)	0.15
35–44	5.16 (0.43, 9.9)	0.03
45–49	5.42 (−0.34, 11.18)	0.07
Residence region		
Middle	Reference	Reference
North	−2.42 (−3.58, −1.25)	< 0.001
South	−2.21 (−3.92, −0.5)	0.01
Number of births	−1.26 (−2.42, −0.11)	0.03
Infertility issues, yes	2.2 (0.59, 3.81)	0.007
Planned hospital for delivery		
Public	Reference	Reference
Military	1.53 (−0.02, 3.08)	0.05
University	1.98 (0.39, 3.58)	0.02
Private	1.24 (−0.28, 2.76)	0.11
Multiple	2.7 (−0.57, 5.98)	0.11

Regional variations were also observed. Women living in the northern and southern regions had significantly lower preferences toward CS compared with those in the middle region, with estimates of −2.42 (95% CI: −3.58 to −1.25; *p* < 0.001) and −2.21 points (95% CI: −3.92 to −0.5; *p* = 0.01), respectively.

Obstetric history also influenced preferences. Each additional birth was associated with a decrease in CS preference by 1.26 points (95% CI: −2.42 to −0.11; *p* = 0.03). Women who had experienced infertility issues were more likely to prefer CS, with an average increase of 2.2 points (95% CI: 0.59–3.81; *p* = 0.007).

The hospital planned for delivery was another key predictor. Women intending to deliver at university hospitals had a significantly higher preference for CS by 1.98 points (95% CI: 0.39–3.58; *p* = 0.02) compared with those delivering in public hospitals. Those planning to deliver in military hospitals showed a borderline significant increase in CS preference (1.53 points; 95% CI: −0.02–3.08; *p* = 0.05). No statistically significant differences were observed among women planning to deliver in private or multiple hospital types.

### 4.6. Prediction of Beliefs Regarding Preferred Mode of Delivery

Regression analysis was performed to determine the predictors of women′s belief systems regarding their preferred mode of delivery. As shown in Table 5, women whose husbands were employed in the private sector had significantly stronger belief alignment toward delivery methods, with an average increase of 2.59 points (95% CI: 1.03–4.14; *p* = 0.001) compared with women whose husbands were unemployed. However, no statistically significant differences in belief scores were found among women whose husbands worked in nonprofit private (*p* = 0.58) or public sectors (*p* = 0.15) relative to the unemployed group.

**Table 5:** Prediction of beliefs regarding preferred mode of delivery among Jordanian women (*n* = 378).

**Variable**	**Estimate (95% CI)**	**p** **value**
Husband employment status		
Unemployed	Reference	Reference
Nonprofit private	0.96 (−2.46, 4.38)	0.58
Public	1.07 (−0.4, 2.54)	0.15
Private	2.59 (1.03, 4.14)	0.001
Insurance coverage type		
Public	Reference	Reference
Military	−0.36 (−1.77, 1.05)	0.61
University	−2.32 (−3.99, −0.65)	0.006
Private	−1.14 (−2.48, 0.21)	0.1
No insurance	−1.78 (−3.22, −0.33)	0.02
Planned hospital for delivery		
Public	Reference	Reference
Military	−0.37 (−1.95, 1.21)	0.65
University	1.46 (0.24, 2.68)	0.02
Private	−0.81 (−1.94, 0.32)	0.16
Nonprofit	−0.18 (−2.52, 2.17)	0.88

Insurance status was also a key factor influencing belief scores. Compared with women with public insurance (reference group), those with university insurance had significantly lower belief alignment scores, with an estimated decrease of −2.32 points (95% CI: −3.99 to −0.65; *p* = 0.006). Similarly, women with no insurance also exhibited lower scores by −1.78 points (95% CI: −3.22 to −0.33; *p* = 0.02). There were no significant differences in belief scores for women with military (*p* = 0.61) or private insurance (*p* = 0.10) compared with those with public insurance.

Hospital choice for delivery also emerged as a significant factor. Women who planned to deliver at university hospitals demonstrated significantly higher belief alignment scores, with an increase of 1.46 points (95% CI: 0.24–2.68; *p* = 0.02) relative to those opting for public hospitals. On the other hand, delivering in military, private, or nonprofit hospitals did not significantly influence belief scores (*p* = 0.65, *p* = 0.16, and *p* = 0.88, respectively).

### 4.7. Prediction of Decision‐Making Among Primiparous Women

To identify the factors influencing decision‐making autonomy among primiparous women, an initial ANOVA was conducted to evaluate the effect of participant characteristics on decision‐making scores. The analysis revealed a significant influence of residence region (*F*(2, 201) = 7.656; *p* < 0.001), whereas no other demographic or obstetric variables were statistically significant.

Subsequently, multiple regression analysis was performed, incorporating region of residence, knowledge about delivery methods, and preference toward normal delivery as predictors. The findings indicated that living in the southern region significantly enhanced women′s involvement in decision‐making, with an estimated increase of 4.00 points (95% CI: 2.37–5.64; *p* < 0.001) compared with those in the middle region (reference group). In contrast, residence in the northern region was not associated with any significant change in decision‐making participation (*p* = 0.85).

Increased knowledge about modes of delivery was linked to a modest yet statistically significant rise in decision‐making involvement, with a coefficient of 0.09 (95% CI: 0.00–0.18; *p* = 0.04). Additionally, a stronger preference toward vaginal delivery predicted a higher likelihood of participation in decision‐making, with an increase of 0.27 points (95% CI: 0.09–0.45; *p* = 0.003).

### 4.8. Prediction of Decision‐Making Among Para‐One Pregnant Women

A preliminary ANOVA was conducted to examine the influence of individual participant characteristics on decision‐making scores among para‐one women. The analysis revealed no statistically significant associations between any demographic or obstetric variables and decision‐making outcomes in this group. Consequently, these variables were excluded from the final regression model.

The subsequent regression analysis identified beliefs about childbirth as the sole significant predictor of decision‐making participation. Specifically, women with stronger positive beliefs about their preferred mode of delivery demonstrated significantly greater involvement in the decision‐making process, with an estimated increase of 0.59 points (95% CI: 0.40–0.77; *p* < 0.001).

## 5. Discussion

This study offered valuable insights into the factors shaping Jordanian women′s preferences, beliefs, and decision‐making regarding their mode of delivery. The findings bear important implications for both maternal health practice and public policy. The participant characteristics and their clinical profiles—along with their sources of information—were examined to understand how these factors influence childbirth choices.

Consistent with previous research [12–14], this study found that doctors and nurses remain the most trusted sources of maternal health information. This reliance reflects a high level of trust in healthcare professionals. However, the increasing use of digital platforms, including the internet and social media, highlights the need to scrutinize the credibility of online information. Ojo et al. [15] emphasized that women often opt for CS based on prior birth trauma, fear of labor pain, cultural beliefs, or the influence of health providers—sometimes without sufficient medical justification. Other studies support this trend, noting that such decisions can be misinformed due to gaps in women′s understanding of delivery modes [16–19].

The findings also revealed a substantial reliance on public healthcare services, with most women planning to deliver in government hospitals. This indicates that affordability and accessibility play major roles in maternal care decisions. This is consistent with the wider literature, particularly in LMICs, where economic constraints influence access and preferences.

In line with previous studies [20, 21], women in the present study displayed moderate knowledge about modes of delivery. However, regional disparities were evident: Women in northern Jordan had significantly higher knowledge scores than those in the central and especially southern regions. This suggests possible inequities in health information dissemination, access to prenatal care, or healthcare infrastructure. Women with high‐risk pregnancies and those receiving care in university‐affiliated facilities also had greater knowledge—likely due to more frequent medical interactions, better patient–provider communication, or structured antenatal education. These insights highlight the importance of integrating comprehensive education programs into routine prenatal care, especially in underserved regions.

Differences in delivery preferences were associated with insurance type and residence region. Women with university or no insurance showed lower preference scores for vaginal delivery than those with public insurance, possibly reflecting unequal access to education, counseling, or facility quality. Healthcare providers must be trained to deliver personalized counseling that considers such demographic and socioeconomic variations. Tailored education should emphasize the benefits and risks of both vaginal and CS delivery and equip women—especially primiparous or high‐risk mothers—with information that supports informed, autonomous decision‐making [22].

The findings also revealed a moderate level of belief alignment regarding delivery preferences, with women whose husbands worked in the private sector showing stronger beliefs—likely linked to greater financial stability. This aligns with other work from Arab countries [23], highlighting how socioeconomic factors intersect with healthcare perceptions. Moreover, women with university or no insurance expressed less confidence in their beliefs, indicating potential inequities in maternal health literacy. Interestingly, those planning to deliver in university hospitals exhibited more favorable beliefs—possibly reflecting perceived higher quality of care or greater exposure to health education.

In terms of decision‐making, women from the southern region were more likely to participate actively in choosing their delivery method. This suggests that regional norms, accessibility, or community health interventions may play a role. Increased knowledge and a positive preference toward vaginal delivery were also significant predictors of decision‐making among primiparous women, echoing the importance of empowerment through education [11]. Conversely, for para‐one women, only personal beliefs were significant predictors of decision‐making autonomy. This underlines the critical need for belief‐centered counseling to counter misconceptions and reinforce an accurate understanding of delivery options.

Together, these findings indicate that individual perceptions, not just medical indications, strongly shape maternal healthcare decisions. Hence, there is an urgent need for belief–informed educational interventions, especially during prenatal care, to build confidence and support well‐informed, values‐aligned choices. Such programs should be adapted to address the specific concerns of para‐one and primiparous women, with an emphasis on trust, cultural context, and equity in information access.

## 6. Strengths and Limitations

This study′s strengths include its robust sample size and diversity—with participants drawn from various regions and healthcare sectors across Jordan. This broad representation enhances the generalizability of the findings. Additionally, the psychometrically validated data collection tool demonstrated good reliability, offering confidence in the consistency of the results.

However, several limitations should be acknowledged. First, the study focused on women′s perceptions, not actual birth outcomes, which limits the ability to assess clinical consequences. Second, the use of self‐administered questionnaires introduces the risk of recall bias and social desirability bias, potentially affecting data accuracy. Third, although the sample was diverse, it excluded some marginalized groups such as Bedouin communities, refugees, and non‐Jordanian residents, limiting the scope of the conclusions. Future research should explore these populations and employ longitudinal designs to assess how women′s knowledge, beliefs, and preferences evolve across the pregnancy‐to‐postpartum continuum.

## 7. Conclusion

This study highlights the critical need to address the preferences, beliefs, and knowledge of Jordanian women concerning their mode of delivery. Findings emphasize the importance of empowering women with accurate, evidence‐based information through culturally sensitive educational initiatives integrated into routine prenatal care. Ensuring equitable access to high‐quality maternal health services, particularly across regions and healthcare sectors, can foster more informed and autonomous decision‐making. To promote safe and satisfying childbirth experiences, coordinated efforts are required from healthcare professionals and policymakers to bridge knowledge gaps, dispel misconceptions, and support women in making informed choices aligned with the best medical practices and personal values. Ultimately, such strategies are vital for improving maternal and neonatal health outcomes in Jordan and comparable contexts.

## Ethics Statement

Ethical approval was received by the Institutional Review Boards of the University of Jordan (10/2024/2867) and the Ministry of Health IRB (Ref: MOHIRB 6/141/2024).

## Disclosure

The contents/findings of this manuscript are the sole responsibility of University Research Co. LLC (URC) and do not necessarily reflect the views of USAID or the United States Government.

## Conflicts of Interest

The authors declare no conflicts of interest.

## Funding

This study was supported by Jordan USAID (10.13039/100021898).

## Data Availability

Data sharing is not applicable to this article, as no datasets were generated or analyzed during the current study.
